# Supplementary material for: MicroRNA-223 Dampens Pulmonary Inflammation during Pneumococcal Pneumonia
Source: Cells. 2023 Mar 21;12(6):959. doi: 10.3390/cells12060959 (PMC10047070; doi:10.3390/cells12060959)
Supplement: Supplementary file 1 [file cells-12-00959-s001.zip › cells-2245411-supplementary.pdf]

cells MDPI

Supplementary Materials

MicroRNA-223 Dampens Pulmonary Inflammation during Pneumococcal Pneumonia

Cengiz Goekeri [1,2,*], Peter Pennitz [1], Wibke Groenewald [1], Ulrike Behrendt [1], Holger Kirsten [3], Christian M. Zobel [4], Sarah Berger [1], Gitta A. Heinz [5], Mir-Farzin Mashreghi [5,6], Sandra-Maria Wienhold [1], Kristina Dietert [7,8], Anca Dorhoi [9,10], Achim D. Gruber [7], Markus Scholz [3], Gernot Rohde [11,12,13], Norbert Suttorp [1,12,14], CAPNETZ Study Group [12], Martin Witzenrath [1,12,14,†] and Geraldine Nouailles [1,*,†]

1 Department of Infectious Diseases, Respiratory Medicine and Critical Care, Charité—Universitätsmedizin Berlin, Corporate Member of Freie Universität Berlin and Humboldt-Universität zu Berlin, 10117 Berlin, Germany
2 Faculty of Medicine, Cyprus International University, 99040 Nicosia, Cyprus
3 Institute for Medical Informatics, Statistics, and Epidemiology, Universität Leipzig, 04107 Leipzig, Germany
4 Department of Internal Medicine, Bundeswehrkrankenhaus Berlin, 10115 Berlin, Germany
5 Therapeutic Gene Regulation, Deutsches Rheuma-Forschungszentrum Berlin (DRFZ), ein Institut der Leibniz-Gemeinschaft, 10117 Berlin, Germany
6 Berlin Institute of Health at Charité—Universitätsmedizin Berlin, BIH Center for Regenerative Therapies (BCRT), 13353 Berlin, Germany
7 Institute of Veterinary Pathology, Freie Universität Berlin, 14163 Berlin, Germany
8 Veterinary Centre for Resistance Research (TZR), Freie Universität Berlin, 14163 Berlin, Germany
9 Institute of Immunology, Friedrich-Loeffler-Institut, 17493 Greifswald, Germany
10 Faculty of Mathematics and Natural Sciences, University of Greifswald, 17489 Greifswald, Germany
11 Department of Respiratory Medicine, Medical Clinic I, Goethe-Universität Frankfurt am Main, 60596 Frankfurt am Main, Germany
12 CAPNETZ STIFTUNG, 30625 Hannover, Germany
13 Biomedical Research in Endstage and Obstructive Lung Disease Hannover (BREATH), German Center for Lung Research (DZL), 30625 Hannover, Germany
14 German Center for Lung Research (DZL), 10117 Berlin, Germany
* Correspondence: cengiz.goekeri@charite.de (C.G.); geraldine.nouailles@charite.de (G.N.)
† The members of the CAPNETZ Study Group are stated in the Acknowledgements section.
‡ These authors contributed equally to this work.

Table S1. Pathway enrichment analysis of pulmonary neutrophils in *S.pn.*-infected miR-223$^{-/-}$ mice.

Term Name	*p*-Value Adjusted	Expected pct	Observed pct	Enrichment
Antimicrobial humoral immune response mediated by antimicrobial peptide	0.0048	0.004	0.093	23.25
Humoral immune response	0.0068	0.01	0.125	12.5
Ribosome	5.75×10^{-9}	0.026	0.266	10.2
Coronavirus disease – COVID-19	1.77×10^{-8}	0.037	0.297	8.02
Peptide biosynthetic process	0.002	0.07	0.297	4.24
Response to bacterium	0.038	0.06	0.25	4.17
Translation	0.0086	0.07	0.28	4
Amide biosynthetic process	0.0078	0.077	0.297	3.86
Peptide metabolic process	0.03	0.085	0.297	3.49
Biological process involved in interspecies interaction between organisms	0.02	0.139	0.39	2.81

Pathway enrichment analysis of granulocyte genes significantly differentially expressed (minimum fold change 1.3) between WT and miR-223$^{-/-}$ mice 24 h following *S.pn.* ST2 infection. GO:BP: Gene

Ontology:Biological Process, KEGG: Kyoto Encyclopedia of Genes and Genomes, pct: percent foreground genes.

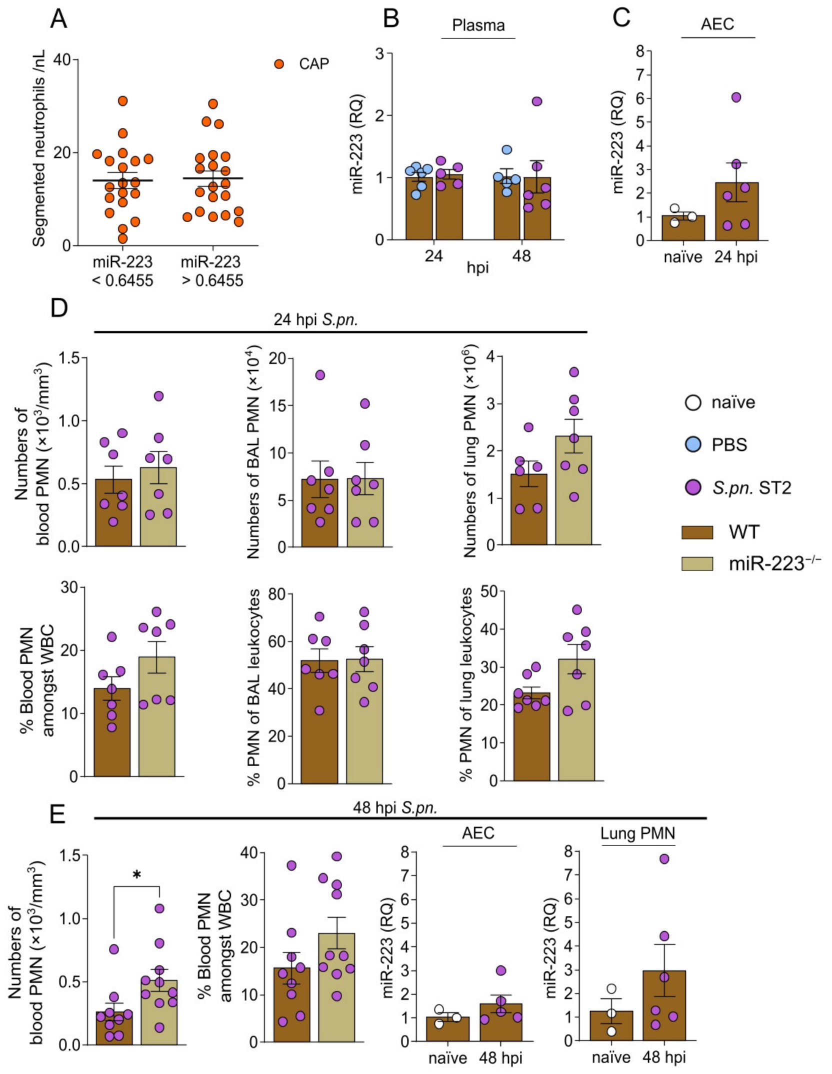

Figure S1. Neutrophil numbers in CAP patients, infected mice and expression of miR-223 in mice plasma, alveolar epithelial cells and lung neutrophils. (**A**) Total numbers of segmented neutrophils recorded in the blood of CAP patients in which serum miR-223 was detected below or above the median relative abundance value of 0.6455 (n = 19 and n = 20, respectively). (**B**) miR-223 expression in the plasma of sham- and *S.pn.*-infected WT mice 24 and 48 hpi (n = 5–6). (**C**) miR-223 expression in AEC of WT naïve (n = 3) or *S.pn.* ST2-infected (n = 6) mice (24 hpi). (**D**) Total numbers and frequencies of blood, BAL and lung PMN in WT and miR-223$^{-/-}$ mice, 24 hpi (n = 6–7). (**E**) Total numbers and frequencies of *S.pn.* ST2-infected blood PMN in WT and miR-223$^{-/-}$ mice, 48 hpi (n = 9–10). (E) miR-223 expression in AEC and lung PMN of WT naïve (n = 3) or *S.pn.* ST2-infected (n = 5–6) mice (48 hpi). Data display individual values and means, error bars represent SEM. (**E**) Mann-Whitney U test, * $p < 0.05$. CAP: community acquired pneumonia, *S.pn.* ST2: *Streptococcus pneumoniae* serotype 2, RQ: relative quantification, hpi: hours post-infection, BAL: bronchoalveolar lavage, AEC: alveolar epithelial cells, WBC: white blood cells, PMN: polymorphonuclear neutrophils.

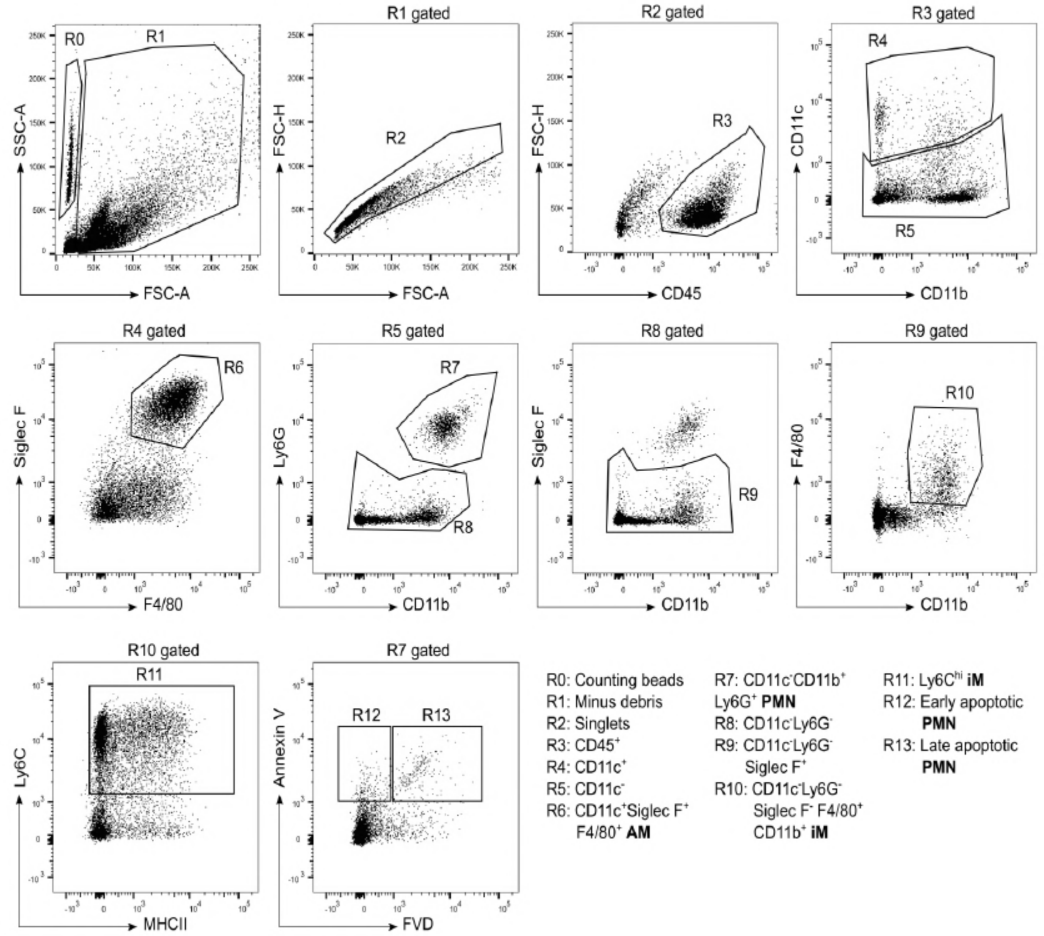

Figure S2. Gating strategy utilized to identify innate leukocyte populations via flow cytometry. AM: alveolar macrophages, iM: inflammatory monocytic macrophages, PMN: polymorphonuclear neutrophils, FVD: fixable viability dye, SSC: side scatter, FSC: forward scatter.

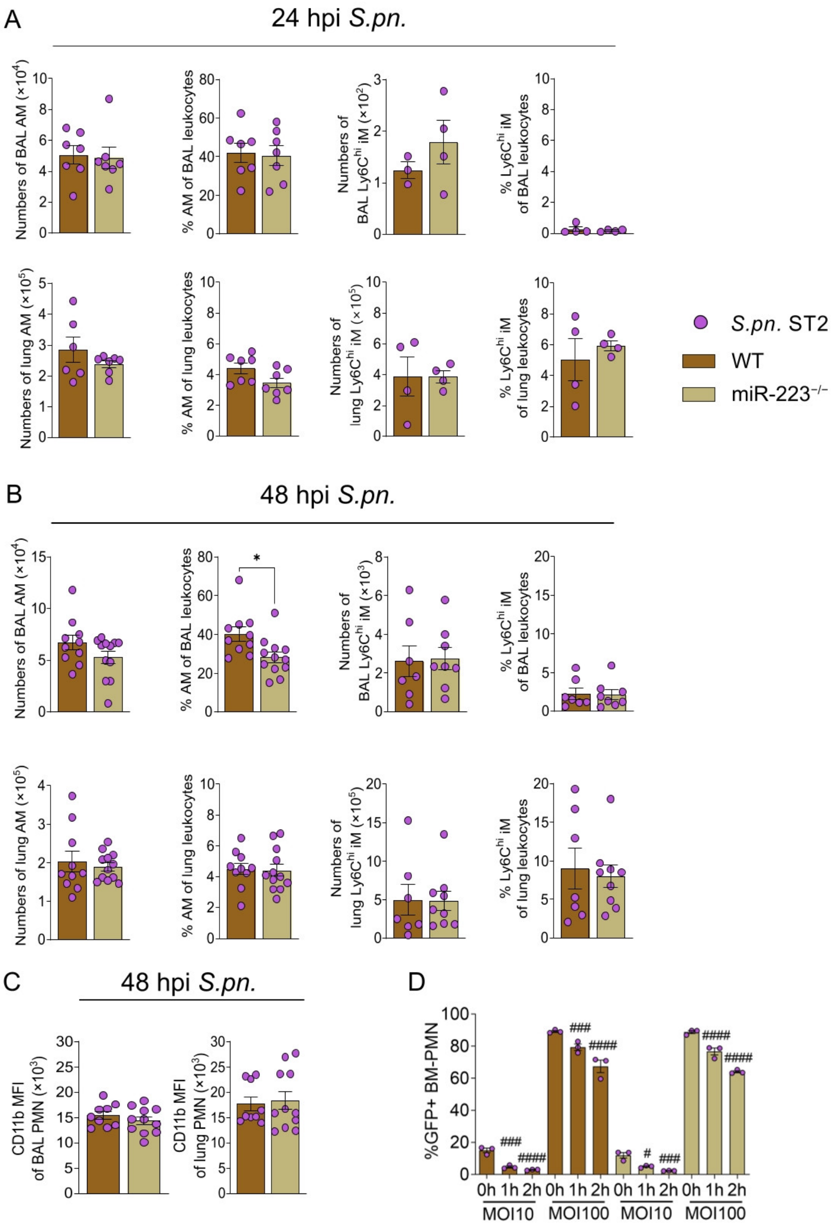

Figure S3. Innate leukocyte populations recorded in BAL and lung tissue of infected mice, MFI analysis of CD11b and neutrophil phagocytosis and killing assay. (**A**) Total numbers and frequencies of *S.pn.* ST2-infected BAL and lung AM and Ly6C^{hi} iM in WT and miR-223$^{-/-}$mice 24 hpi (n = 3–7). (**B**) Total numbers and frequencies of *S.pn.* ST2-infected BAL and lung AM and Ly6C^{hi} iM in WT and miR-223$^{-/-}$ mice 48 hpi (n = 7–12). (**C**) MFI analysis of CD11b in BAL and lung PMN of *S.pn.* ST2-infected WT and miR-223$^{-/-}$mice 48 hpi (n = 9–11). (**D**) Phagocytosis and killing assay of WT and miR-223$^{-/-}$BM-PMN. BM-PMN were stimulated with GFP-expressing *S.pn.* ST2 and evaluated for phagocytosis (0h) and killing (1 h, 2 h) of *S.pn.* ST2. WT and miR-223$^{-/-}$mice (n = 3) were sacrificed and the BM-PMN isolated and plated in triplicate for each condition and time point. (**B**) Unpaired t-test, * $p < 0.05$. (**D**) A hash sign (#) denotes statistical difference between the *S.pn.*-infected groups (0 h vs 1 h, 0 h vs 2 h) at the corresponding MOI. # denotes $p < 0.05$ (2-way ANOVA/Sidak's multiple comparisons test), ### denotes $p < 0.001$ (2-way ANOVA/Sidak's multiple comparisons test), #### denotes $p < 0.0001$ (2-way ANOVA/Sidak's multiple comparisons test). Data display means and individual values. Error bars represent SEM. *S.pn.* ST2: *Streptococcus pneumoniae* serotype 2, hpi: hours post-infection, BAL: bronchoalveolar lavage, AM: alveolar macrophages, Ly6C^{hi} iM: Ly6C^{hi} inflammatory monocyte-derived macrophages, BM-PMN: bone marrow

neutrophils, GFP: green fluorescent protein, MFI: mean fluorescence intensity, MOI: multiplicity of infection.

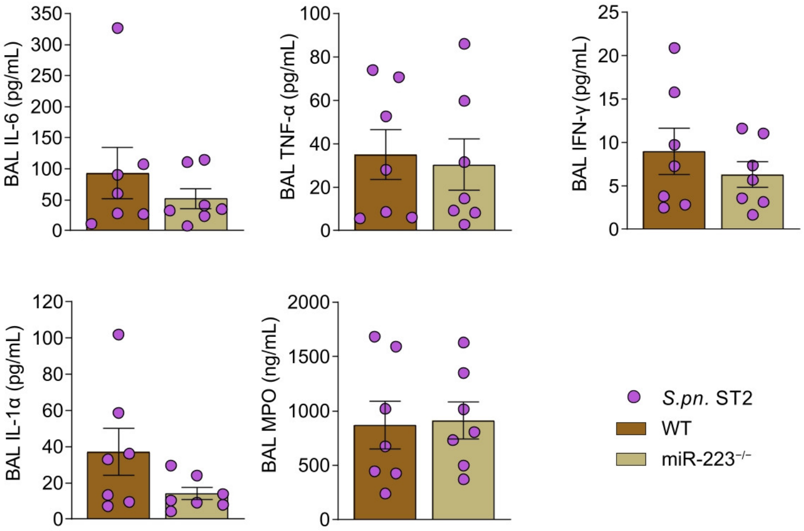

Figure S4. Pro-inflammatory cytokine and chemokine production in WT and miR-223$^{-/-}$ mice 24 hpi. Pro-inflammatory cytokines were quantified in the BAL of WT and miR-223$^{-/-}$mice 48 hpi (*n* = 7) using the LEGENDPlex Mouse Inflammation Panel (BioLegend, San Diego, CA, USA), whilst MPO was quantified using the MPO Mouse ELISA kit (Hycult Biotech, Uden, The Netherlands). Data display individual values and means, error bars represent SEM. BAL: bronchoalveolar lavage, *S.pn.* ST2: *Streptococcus pneumoniae* serotype 2, MPO: myeloperoxidase.

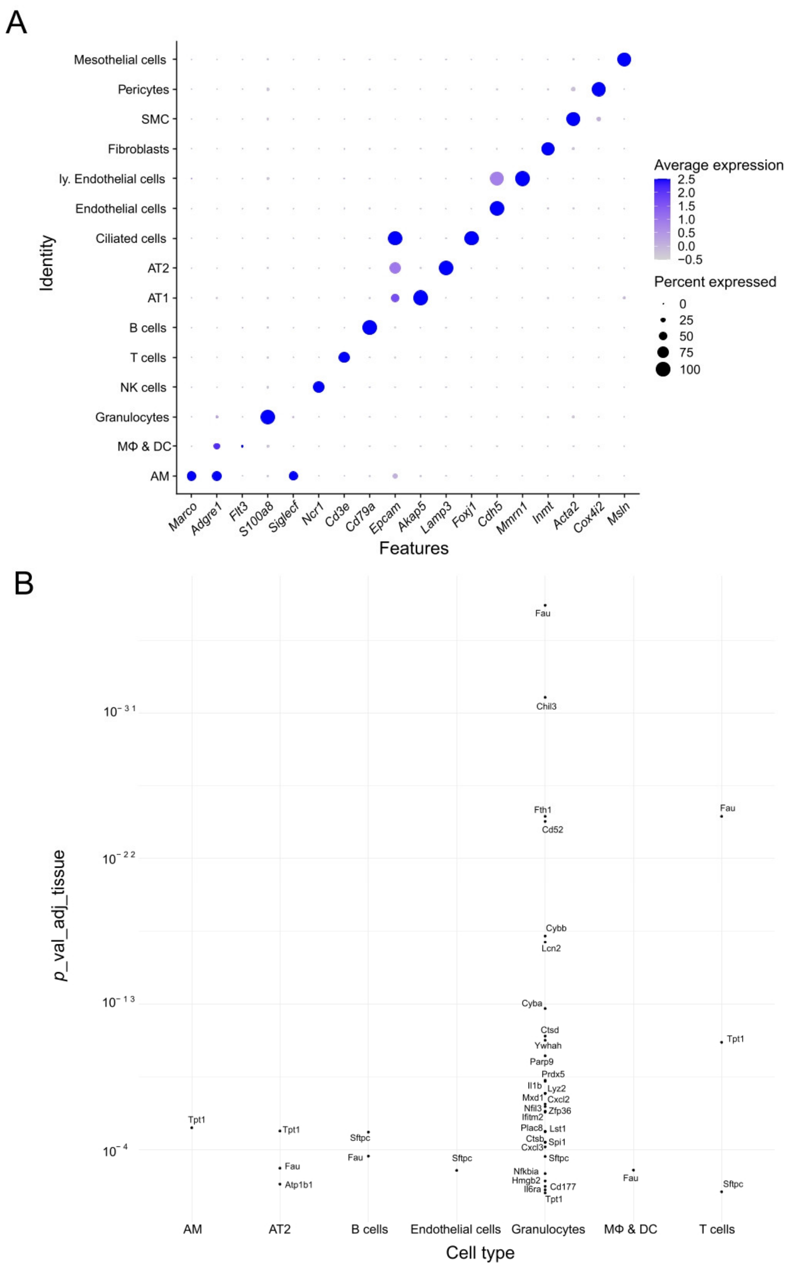

Figure S5. Identification of cell clusters and *p*-values of differentially expressed granulocyte genes in scRNA-seq of *S.pn.*-infected miR-223$^{-/-}$ murine lung cells. (**A**) Dot plot of marker genes used to identify cellular subsets following scRNA-seq. Coloration and point sizes indicate average expression and percent expressed, respectively. (**B**) Scatter plot illustrating *p*-values of differentially expressed genes in lung cells of *S.pn.* ST2-infected miR-223$^{-/-}$ mice. AT1: alveolar epithelial cells type I, AT2: alveolar epithelial cells type II, AM: alveolar macrophages, SMC: smooth muscle cells, MΦ & DC: interstitial and inflammatory macrophages/monocytes and dendritic cells, ly. Endothelial cells: lymphatic endothelial cells, NK cells: natural killer cells, *p*_val_adj_tissue: *p*-values adjusted by the Bonferroni multiple testing correction for the identified cell types and genes.

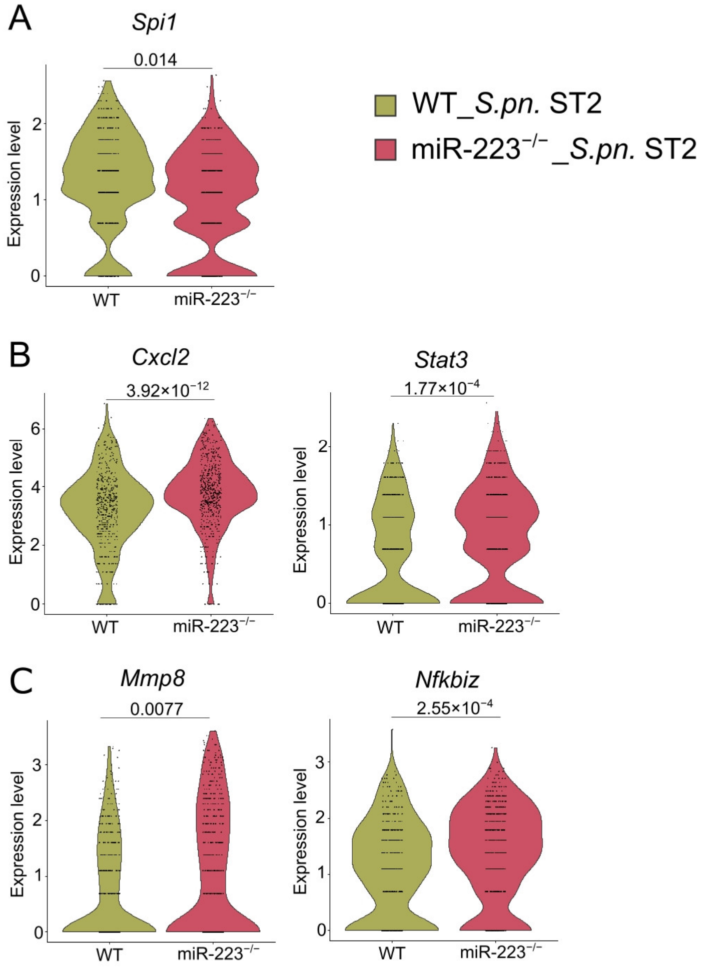

Figure S6. Violin plots displaying the expression level of differentially expressed genes in granulocytes of *S.pn.* ST2-infected WT and miR-223$^{-/-}$ mice. Differential gene expression was exhibited in (**A**) an inducer of miR-223 (*Spi1*), (**B**) validated miR-223 targets (*Cxcl2, Stat3*) and (**C**) inflammatory mediators (*Mmp8*, *Nfkbiz*). Adjusted *p*-values displayed above violin plots.
